# A tumor-microenvironment-responsive nanomaterial for cancer chemo-photothermal therapy†

**DOI:** 10.1039/d0ra04171h

**Published:** 2020-06-09

**Authors:** Kaiyu Wang, Zhiyuan Cai, Rong Fan, Qian Yang, Tao Zhu, Zhongying Jiang, Yuqiang Ma

**Affiliations:** Department of Physics, National Laboratory of Solid State Microstructures, Collaborative Innovation Center of Advanced Microstructures, Nanjing University Nanjing China zhuttd@nju.edu.cn myqiang@nju.edu.cn; Key Laboratory of Micro-nano Electric Sensing Technology and Bionic Devices, College of Electronic and Information Engineering, Yili Normal University Yining China jiangzhying@163.com

## Abstract

Taxol (TAX) is a typical anticancer drug that is widely used in clinical treatment of cancer, while gold nanorods (AuNRs) are a kind of well-known material applied for photothermal therapy (PTT). The therapeutic outcome of TAX in chemotherapy is however limited by drug resistance, while AuNRs often show poor accuracy in PTT. To optimize the functions of TAX and AuNRs, we developed a hydrogen peroxide (H_2_O_2_)-triggered nanomaterial (LV–TAX/Au@Ag) for combined chemo-photothermal therapy. In normal tissues, TAX is protected in the lipid bilayer and isolated from the surrounding normal cells, while AuNRs are coated with silver shells and show low photothermal capacity. However, after reaching the tumor tissues, the silver shells can be etched by endogenous H_2_O_2_ in the tumor microenvironment, and the photothermal properties of AuNRs are then recovered. Meanwhile, the generated oxygen destabilizes the LV, which makes the 100 nm sized nanosystems disassemble into the smaller sized TAX and AuNRs, leading to the deep penetration and direct interaction with tumor tissues. The related *in vitro* experiments proved the validity of this “turn off/on” effect. Extensive necrosis and apoptosis were observed in the tumor tissues and the proliferation of solid tumor was greatly suppressed due to this combined chemo-photothermal therapy. In addition, no significant damage was found in normal tissues after the treatment of LV–TAX/Au@Ag. Therefore, the strategy to achieve environmental response by modifying the photothermal agents enhanced the efficiency and safety of nanomedicine, which may help improve cancer treatment.

## Abstract 2

Endogenous hydrogen peroxide was utilized to control the release of agents for better tumor therapeutic effect and safety.

## Graphical abstract

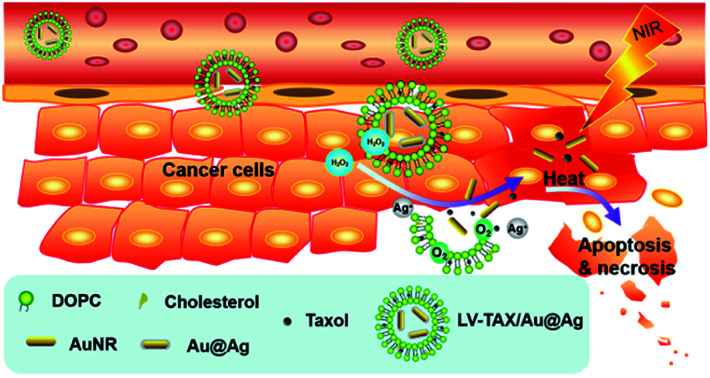

## Introduction

Cancer has always been the focus of people's attention due to its horrific fatality rate.^1,2^ Chemotherapy is the most typical and conventional way that is widely applied to cancer treatment in the clinic.^3^ Nanocarriers like liposomes are then developed for prolonged circulation life and thus better therapeutic effect.^4–9^ The main components of liposomes are phospholipids and cholesterol, which are of high biocompatibility due to the similar structure with biological membranes. The hydrophobic drugs can be loaded into the lipid bilayers while the hydrophilic drugs can be loaded into a liquid core of liposomes. Moreover, liposomes with the size of about one hundred nanometers are able to escape from the arrest by the reticulo-endothelial system (RES) due to the hydrophilic surface and can be further targeted to the tumor tissues *via* the EPR effect.^10–13^

However, the release of drugs after the arrival at the tumor tissues is still a big challenge. The specific characteristics such as low pH and hypoxia in tumor microenvironment are utilized by researchers with the aim of controlled-release.^14,15^ Liposomes composed of pH-sensitive lipids or fabricated with pH-sensitive polymers are stable in normal tissues and destabilize under acidic conditions.^16–18^ Liposomes conjugated with hypoxia-sensitive molecule like nitroimidazole also have the tendency to disintegrate due to the change of water solubility under hypoxic conditions caused by the transformation from nitroimidazole to aminoimidazole.^19,20^ Therefore, the pH-responsive or hypoxia-responsive liposomes could release drugs only after reaching at the tumor sites. Besides, other drug release triggers such as light and temperature are also utilized in drug delivery systems with controlled-release.^9,21–23^

Although the drug delivery systems with controlled-release can deliver the anticancer drugs to the tumor tissues to a great extent, the current chemotherapy still remains unsatisfying because of multiple drug resistance (MDR). The combination of chemotherapy with photothermal therapy (PTT) may be a promising strategy to improve the therapeutic effect in recent years as the hyperthermia or induced by near-infrared light can directly kill cancer cells.^24,25^ As previously reported, a single round of PTT combined with a sub-therapeutic dose of drugs could elicit robust anti-tumor immune responses and eliminate local as well as untreated, distant tumors in >85% of animals bearing CT26 colon carcinoma.^26^ However, how to realize the combination therapy efficiently and securely is still a big challenge.

To achieve the combined chemo-photothermal therapy, researchers have taken advantages of the surface-modifiable photothermal materials to load anticancer drugs, such as loading doxorubicin (DOX) into nanographene oxide or conjugating curcumin on the surface of AuNRs.^27,28^ Considering the protection of drugs, many researchers have also tried to wrap chemical drugs and photothermal agents together in the nanocarriers. For example, Zheng *et al.* have used a single-step sonication method to load doxorubicin (DOX) and indocyanine green (ICG) in the self-assembled polymer nanoparticles for combination therapy. The combined treatment not only synergistically induced the apoptosis and death of DOX-sensitive MCF-7, but also showed great cytotoxicity to DOX-resistant MCF-7/ADR cells.^25^ Similarly, Cao *et al.* have also integrated DOX, ICG, and gadolinium(iii)-chelated silica nanospheres to form a theranostic platform including chemotherapy, PTT, and magnetic resonance imaging (MRI). The complex could effectively improve the therapeutic efficacy compared to the single treatment and showed the ability to be the T_1_-type MRI contrast agents.^29^ However, these have not fully developed the potential of photothermal agents. Moreover, the simple physical encapsulation can not affect the property of photothermal agents, suggesting that the normal tissues around the material may be also damaged under irradiation. The security of treatment is thus compromised greatly.

**Fig. 1:**
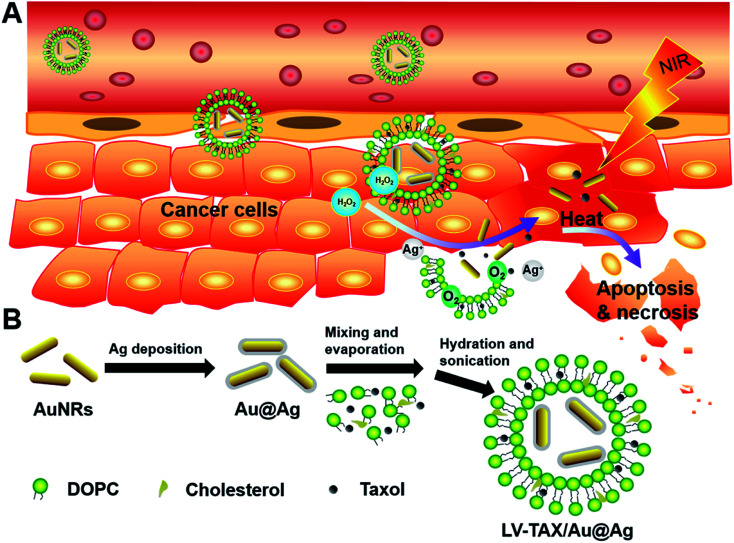
(A) Combination therapy of LV–TAX/Au@Ag was turned on by the endogenous H_2_O_2_ in tumor microenvironment under near-infrared irradiation. (B) Synthetic process of LV–TAX/Au@Ag.

Herein, we developed a tumor-microenvironment-responsive nanomaterial (LV–TAX/Au@Ag) for combination therapy, whose chemotherapy and PTT were turned on by the endogenous hydrogen peroxide (H_2_O_2_). As shown in Fig. 1, the photothermal agent gold nanorods (AuNRs) were first coated with silver (Au@Ag) and then encased in the liquid core of lipid vesicles (LV), while the anticancer drug taxol (TAX) was loaded in the hydrophobic bilayers of LV. Due to the presence of silver shells, the ability of AuNRs to absorb near-infrared light was reduced significantly. Meanwhile, the toxicity of TAX was also suppressed because of the LV coating. Therefore, the functions of TAX and AuNRs were temporarily inhibited in normal physiological conditions, which meant that LV–TAX/Au@Ag could not cause damage to cells in normal tissues even under near-infrared light. However, when the developed LV–TAX/Au@Ag reached the tumor *via* the EPR effect, the endogenous H_2_O_2_ in tumor microenvironment was then utilized as the trigger of combination therapy through etching the silver at the surface of AuNRs.^30–32^ The silver shells of Au@Ag were etched by H_2_O_2_ and then produced oxygen and silver ions, as well as baring the AuNRs, which recovered the photothermal conversion capacity of AuNRs. Thus, the accuracy of PTT was greatly improved and the damage to normal cells owing to thermo was reduced significantly. Furthermore, the generated oxygen could then rupture LV and result in the release of TAX and AuNRs. The released materials could then further penetrate into the solid tumor due to their smaller size. TAX could inhibit the mitosis of tumor cells, while the bare AuNRs could heat up and further destroyed the tumor cells under 808 nm laser irradiation, leading to an enhanced therapeutic effect compared with the single treatment.^33–35^ Relevant measurements combined with *in vitro* and *in vivo* experiments were carried out to prove that LV–TAX/Au@Ag were able to diagnose and treat the HeLa-tumor-bearing mice efficiently without damaging the normal tissues in the process of combination therapy. Therefore, we successfully combined chemotherapy and PTT through physical encapsulation and surface modification, and suppressed the lethality of chemical drugs and photothermal materials to normal tissues during treatment, thus improving the efficiency and safety of nanomedicine.

## Material and methods

### Materials

Chloroauric acid (HAuCl_4_), cetyl trimethyl ammonium bromide (CTAB), sodium borohydride (NaBH_4_), silver nitrate (AgNO_3_), ascorbic acid, sodium hydroxide (NaOH) were purchased from Macklin Biochemical Technology Co., Ltd. (Shanghai, China). 1,2-Dioleoyl-*sn*-glycero-3-phosphocholine (DOPC), cholesterol and TAX were purchased from Sigma-Aldrich (St Louis, MO, USA). Dulbecco's modified eagle medium (DMEM), calcein-AM, 3-(4,5-dimethyl-2-thiazolyl)-2,5-diphenyl-2-*H*-tetrazolium bromide (MTT), propidiumiodide (PI) and annexin V-fluorescein isothiocyanate (FITC) were purchased from Jiangsu KeyGEN BioTECH Corp., Ltd. (Nanjing, China). Nude mice (female, 20 ± 2 g) were purchased from Animal Central Laboratory of Nanjing Medical University (Nanjing, China). All other chemicals were of analytical degree and purchased from Nanjing Chemical Reagent Co., Ltd. (Nanjing, China).

### Preparation of AuNRs and Au@Ag

AuNRs were synthesized through a seed-mediated growth method as previously reported.^36,37^ Briefly, the whole process was divided into three steps. First, 100 μL of HAuCl_4_ (0.01 M) and 1.88 mL of CTAB (0.2 M) were added to 6.25 mL deionized water. 0.5 mL of NaBH_4_ (0.01 M) was then added to the solution to form the gold seed solution. Next, the growth solution was prepared by adding 5.4 mL of AgNO_3_ (0.01 M) and 5.4 mL of ascorbic acid (0.1 M) to an aqueous solution containing 29.7 mL of HAuCl_4_ (0.01 M), 425.7 mL of CTAB (0.2 M) and 427.5 mL deionized water. At last, 7.2 mL of gold seed solution was added to the mixture solution and was kept at 38 °C overnight. The reaction solution was centrifuged at 12 000 rpm for 20 min and washed with deionized water for three times to purify the prepared AuNRs. The purified AuNRs were lyophilized and stored at 4 °C for further usage. In order to obtain Au@Ag, the modified procedure was used as previously reported.^38,39^ Herein, 0.1 mL of AgNO_3_ (0.01 M) and 50 μL of ascorbic acid (0.1 M) were added to 50 mL of CTAB (0.1 M) solution containing 1 mg of AuNRs. 0.5 mL of NaOH (0.1 M) was then added to the mixture to increase the pH over 10 and was vigorously stirred for 1 h to ensure the complete reduction reaction. Finally, the reactant was also centrifuged at 12 000 rpm for 20 min and washed with deionized water thrice for purification.

### Preparation of LV–TAX/Au@Ag

The surface of Au@Ag was first modified by adding 0.5 mg of prepared Au@Ag to 1 mL of DOPC (0.05 M in chloroform).^7,40^ The mixture was then gently stirred for 2 h at room temperature and collected *via* centrifugation. After that, the mixture of DOPC : cholesterol : TAX (8 : 1 : 1) was dissolved in chloroform at a concentration of 2 mg mL^−1^. Cholesterol was added in order to enhance the rigidity of LV for better efficiency of tumor penetration as previously reported by researchers.^8^ 250 μL of the mixture of in the vial was evaporated under a nitrogen flow to form the lipid film. The resulting lipid film was then kept in a vacuum oven at room temperature overnight to remove any residual chloroform. Afterwards, the lipid film was hydrated in 1 mL of PBS that containing the surface modified Au@Ag under the occasional shake for 2 h. At last, the hydrated solution was sonicated for 20 min and purified *via* centrifugation to acquire the developed LV–TAX/Au@Ag. The encapsulation rate of TAX was sequentially measured by high performance liquid chromatography (HPLC) (Waters, USA).^41^ Moreover, LV–TAX were prepared according to the above steps without the adding of Au@Ag, while LV–Au@Ag were prepared in absence of TAX. LV–AuNRs were obtained by replacing Au@Ag with AuNRs during the synthesis of LV–Au@Ag. The synthetic procedures of LV–TAX/AuNRs were same as that of LV–TAX/Au@Ag through mixing AuNRs instead of Au@Ag with DOPC.

### Characterization

The morphology was examined by a transmission electron microscope (TEM) (JEOL, Japan). The average size was measured by dynamic light scattering (DLS) (Malvern, UK). The surface element composition was investigated by X-ray photoelectron spectroscope (XPS) (PHI 5000 Versa Probe, Japan). The absorption spectra of nanomaterials were measured by ultraviolet-visible (UV-vis) spectrophotometer (YOKE, China).

### *In vitro* drug release

The vitro drug release profiles of LV–TAX/AuNRs and LV–TAX/Au@Ag were measured through a dialysis method as previously reported.^41^ The solution of LV–TAX/AuNRs or LV–TAX/Au@Ag was firstly put in a dialysis bag. The dialysis bag was then incubated in 200 mL of PBS at 37 °C with magnetic stirring. 0.7 mL of incubation medium was taken out at determined time points and 0.7 mL of fresh PBS was supplied accordingly. The collected 0.7 mL of aqueous solution was mixed with 0.3 mL of acetonitrile subsequently. The concentration of TAX in the mixture was finally measured by HPLC. On the other hand, in order to simulate the tumor microenvironment, 200 mL of PBS that contain H_2_O_2_ (10 mM) was used to replace the pure PBS at the beginning.

### *In vitro* photothermal study

The heating profiles of LV–TAX/AuNRs, LV–TAX/Au@Ag and LV–TAX/Au@Ag etched with H_2_O_2_ were measured by a thermometer *in vitro*, as well as PBS in contrast. These materials were first dispersed in PBS respectively as the concentration of Au was 1 mg mL^−1^ uniformly. The solutions were irradiated by an 808 nm laser (1 W cm^−2^) and the real-time temperature was measured every minute. The heating curve of LV–TAX/Au@Ag with different Au concentrations that etched with H_2_O_2_ was also measured subsequently.

### MTT assay

The security of LV–TAX/Au@Ag against human umbilical vein endothelial cells (HUVEC) was measured by MTT assay. HUVEC were seeded in 96-well plates at a density of 10^4^ cells per well in Dulbecco's modified eagle medium (DMEM) and incubated until the confluence of cells reached 80% in each well (37 °C, 5% CO_2_). Then the medium was replaced by 100 μL of fresh medium with different Au concentrations of LV–TAX/Au@Ag. After 4 h, the designated wells were irradiated by an 808 nm laser (1 W cm^−2^) for 6 min. Afterwards, the medium of all wells was removed and cells were washed thrice with PBS (pH = 7.4). Then 20 μL of MTT solution (2.5 mg mL^−1^ in PBS) and 80 μL of culture medium was added into each well, followed by incubating for another 2 h. The medium was then aspirated and 200 μL of dimethylsulfoxide (DMSO) solution was added to each well. After 15 minutes, their absorbance at 490 nm was measured using an iMark Enzyme mark instrument (Bio-Rad Inc., USA). The cell viability was calculated according to previous report.^42^ Moreover, the *in vitro* cytotoxicity of LV–TAX/AuNRs against HUVEC was also measured in the same way as a contrast.

### *In vitro* apoptotic analysis

The *in vitro* cytotoxicity of different materials against HeLa cells was examined by fluorescence imaging and flow cytometry respectively. HeLa cells were seeded in 6-well plates at a density of 10^6^ cells per well in DMEM and incubated until the confluence of cells reached 80% in each well (37 °C, 5% CO_2_). Different materials including PBS, TAX, LV–AuNRs, LV–TAX/Au@Ag (etched with H_2_O_2_) were respectively added to the wells (the Au concentration of both LV–AuNRs and LV–TAX/Au@Ag was 1 mg mL^−1^ while the concentration of TAX was 0.1 mg mL^−1^). PBS was selected as the control group, while the TAX, LV–AuNRs, and LV–TAX/Au@Ag represented chemotherapy, PTT, and combination therapy, respectively. After 12 h, the wells were irradiated by an 808 nm laser for 6 minutes. For fluorescence imaging, HeLa cells were simply dyed with calcein-AM/PI and observed by a fluorescence microscope. The excitation and emission wavelength of calcein-AM was 490 nm and 515 nm, while the excitation and emission wavelength of PI was 535 nm and 617 nm. For flow cytometry, the whole medium and cells in each well were collected. The mixture was dyed with annexin V-FITC/PI and observed by flow cytometer (FCM).

### *In vivo* infrared thermal imaging

Three groups of nude mice that bearing HeLa-tumor were respectively intravenously injected with 100 μL of PBS, AuNRs, and LV–TAX/Au@Ag (1 mg mL^−1^). The mice were then irradiated by an 808 nm laser (1 W cm^−2^) 12 h after injection. Meanwhile, the whole body of each mouse was imaged by using an infrared thermal camera to monitor the change of temperature during the irradiation.

### Bio-distribution study

The HeLa-tumor-bearing mice were intravenously injected with 100 μL of LV–TAX/Au@Ag solution (1 mg mL^−1^). The mice were sacrificed and the tumor and main organs (heart, liver, spleen, lung, and kidney) were extracted 2, 4, 12, 24, 48 h post the injection, respectively. Moreover, the blood was also collected subsequently. After being washed and weighed, the tumor and organs were dissolved by aqua regia solution. The concentration of gold was eventually determined by Inductively Coupled Plasma Optical Emission Spectrometer (ICP-OES) (Optima 5300DV, USA).

### Histological examination

The HeLa-tumor-bearing mice were intravenously injected with 100 μL of LV–TAX/Au@Ag solution (1 mg mL^−1^). Radiation was brought to bear on the mice by an 808 nm laser (1 W cm^−2^) for 6 min after the injection. The mice were sacrificed and the main organs (heart, liver, spleen, lung, and kidney) were extracted 1, 7, 14, 21, 28 d post the treatment, respectively. The organs were then put into 4% paraformaldehyde for at least 7 days. Afterwards, all the organs were made into sections that stained with H&E and observed under the microscope.

### *In vivo* antitumor effect

HeLa-tumor-bearing models were established by challenging nude mice with subcutaneous injection of HeLa cells (10^7^ per mouse) at the right armpits. The mice were randomly divided into five groups (Control, LV–TAX, LV–Au@Ag, LV–TAX/AuNRs, and LV–TAX/Au@Ag) when the tumor volume reached about 500 mm^3^. Afterwards, the mice in five groups were intravenously injected with 100 μL corresponding solutions (the mice in control group were injected with PBS) respectively. Furthermore, the mice were irradiated for 6 min by an 808 nm laser (1 W cm^−2^) 12 h after the injection. The tumor volume and survival number of mice in each group were measured and recorded every two days. The relative tumor volume (*V*/*V*_0_) was then calculated as *V*_0_ represented the initial volume of tumor. After 15 days, the mice were sacrificed and the tumors were collected and stored in 10% neutral buffered formalin solution. At last, the tumors were made into slices and stained with hematoxylin and eosin (H&E), TdT-mediated dUTP nick end labeling (TUNEL) and Ki67 respectively. The stained slices were finally observed and imaged by a fluorescence microscope. All protocols for animal test were reviewed and approved by the committee on animals in Nanjing University and carried on according to guidelines given by National Institute of Animal Care.

### Statistical analysis

All data were analyzed using SPSS 16.0 statistical software (SPSS Inc, Chicago, IL, USA) and are expressed as mean values. Differences between data from the experimental and control groups were analyzed using one-way analysis of variance (ANOVA) to determine statistical significance. *P* < 0.05 was considered statistically significant.

## Results and discussions

### Preparation and characterization of LV–TAX/Au@Ag

**Fig. 2:**
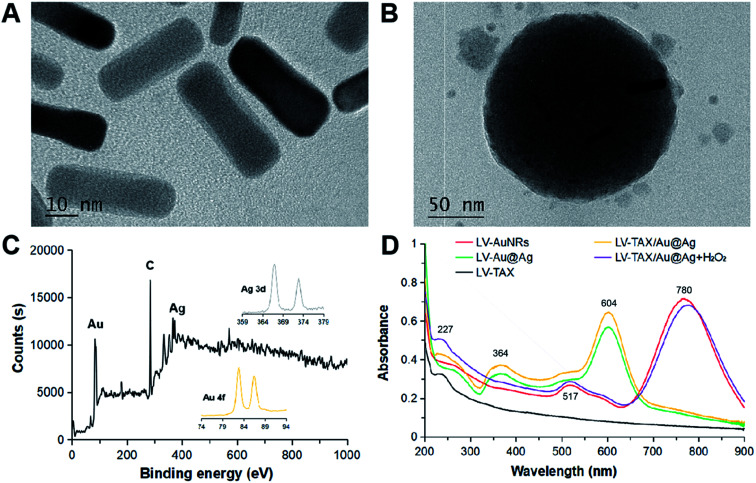
Characterization of LV–TAX/Au@Ag. TEM images of (A) Au@Ag and (B) LV–TAX/Au@Ag. (C) XPS spectrum of LV–TAX/Au@Ag. (D) UV-vis absorption spectra of LV–AuNRs, LV–Au@Ag, LV–TAX, LV–TAX/Au@Ag with and without H_2_O_2_.

AuNRs were first synthesized through a seed-mediated growth method as previously reported.^36,37^ The size and morphology of AuNRs were measured by TEM (Fig. S1A†). The prepared AuNRs were found to be ∼30 nm in length and ∼10 nm in width. After the coating of silver, the size became a little bit larger and the obvious silver shell could be observed by TEM (Fig. 2A). Moreover, the LV and LV–TAX/Au@Ag were respectively prepared and measured by TEM and DLS (Fig. 2B, S1B and S2†). The modified gold nanorods, Au@Ag, could be found in the internal core of LV. The average size of LV–TAX/Au@Ag was 148 ± 12 nm, which was slightly larger than that of LV (140 ± 13 nm). XPS (Fig. 2C) and UV-vis (Fig. 2D) were employed to verify the successful synthesis of LV–TAX/Au@Ag. The peak of Au element and Ag element were both found in the XPS spectrum, which meant the existence of Au@Ag in the core of LV–TAX/Au@Ag. The typical double absorption peaks of AuNRs could be found in the spectra of LV–AuNRs at 517 nm and 780 nm, indicating that the existence of LV did not influence the photothermal conversion capability of AuNRs. After being coated with silver, the double absorption peaks had a blue shift to 364 nm and 604 nm. On the other hand, we could find the typical absorption peak of TAX at 227 nm in the spectra of LV–TAX. Furthermore, as the absorption peaks of Au@Ag and TAX were all shown in the spectrum of LV–TAX/Au@Ag, the successful coating of Au@Ag and loading of TAX were thereby proved. Meanwhile, the encapsulation rate of TAX was measured by HPLC and calculated to be 87.29%. Moreover, after being etched with H_2_O_2_, LV–TAX/Au@Ag had the double absorption peaks close to those of AuNRs instead of Au@Ag, which meant the dissolution of Ag and the restoration of photothermal capacity.

**Fig. 3:**
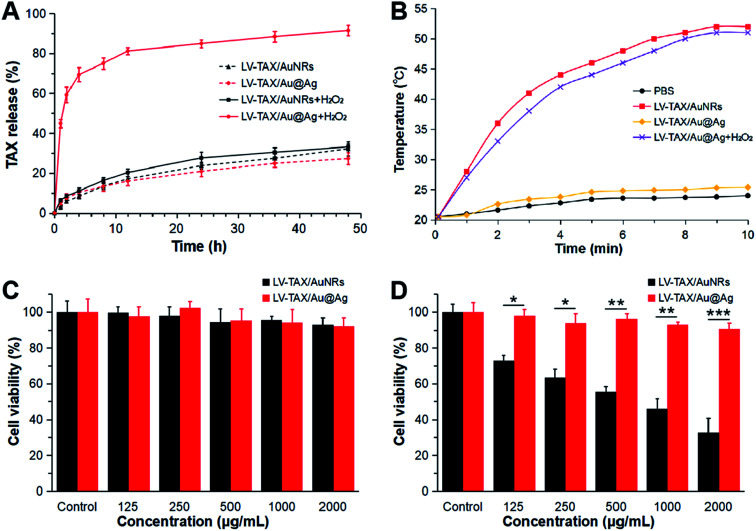
(A) Drug release profiles of LV–TAX/AuNRs and LV–TAX/Au@Ag under different conditions. (B) Temperature elevation curves of PBS, LV–TAX/AuNRs, LV–TAX/Au@Ag, and LV–TAX/Au@Ag etched with H_2_O_2_ exposed to an 808 nm laser (1 W cm^−2^). (C) Cell viability of HUVEC incubated with LV–TAX/AuNRs or LV–TAX/Au@Ag at different concentrations. (D) Cell viability of HUVEC incubated with LV–TAX/AuNRs or LV–TAX/Au@Ag at different concentrations after being irradiated by an 808 nm laser (1 W cm^−2^). All data represent mean ± SD (*n* = 5). *p < 0.05, **p < 0.01, ***p < 0.001.

The drug release of LV–TAX/Au@Ag was then examined (Fig. 3A). As we can see, LV–TAX/Au@Ag exhibited a poor release profile under the normal condition, they only released about 27% of TAX in 48 h, which is similar to LV–TAX/AuNRs, indicating that both of them could only release TAX *via* permeation in normal tissues. However, when LV–TAX/Au@Ag were immersed in the solution that contained excess H_2_O_2_ just like the microenvironment of tumor, they could release TAX rapidly. LV–TAX/Au@Ag released about 80% of TAX in 12 h and over 90% at last, which was much larger than that of LV–TAX/AuNRs as they only released about 33% of TAX in 48 h. This huge contrast suggested that TAX release could be triggered by H_2_O_2_ in the microenvironment of tumor, as the silver shell at the out of AuNRs could react with H_2_O_2_ and generated oxygen, leading to the rupture of LV and thus the huge release of TAX. Afterwards, related heating curves were measured in order to examine the photothermal ability of LV–TAX/Au@Ag (Fig. 3B). The heating curve of LV–TAX/Au@Ag etched with H_2_O_2_ was similar to that of LV–TAX/AuNRs, as the temperature increased to about 45 °C rapidly in 5 min and persistently rose to over 50 °C at last under the irradiation of laser, which was much higher than that of PBS and was enough to kill tumor cells. In contrast, the heating rate of LV–TAX/Au@Ag without H_2_O_2_ was much slower due to the blue shift of absorption peak, indicating that the photothermal efficiency of LV–TAX/Au@Ag was inhibited in normal condition. The photothermal conversion experiment of LV–TAX/Au@Ag (etched with H_2_O_2_) at various concentrations was also carried out to measure the photothermal capacity of LV–TAX/Au@Ag (Fig. S3†). As we can see, LV–TAX/Au@Ag (etched with H_2_O_2_) had the favourable photothermal conversion efficiency. Furthermore, the higher the concentration, the better the performance. At last, the biocompatibility of LV–TAX/Au@Ag was tested by incubating with HUVEC (Fig. 3C). Both LV–TAX/AuNRs and LV–TAX/Au@Ag showed low cytotoxicity to HUVEC, as the cell viability was generally high and was still higher than 90% even when the concentration was up to 2000 μg mL^−1^. However, after the irradiation of an 808 nm laser, the two exhibited entirely different cytotoxicity against HUVEC (Fig. 3D). Cell viability in the group of LV–TAX/Au@Ag still remained at a high level, while a large number of cells died in the group of LV–TAX/AuNRs whose cell viability decreased from 73% to 32% as the concentration increased. Therefore, we can conclude that LV–TAX/Au@Ag showed the negligible cytotoxicity to normal cells even under the irradiation of laser and might be nondestructive in normal tissues during the process of PTT.

### *In vitro* antitumor effect

**Fig. 4:**
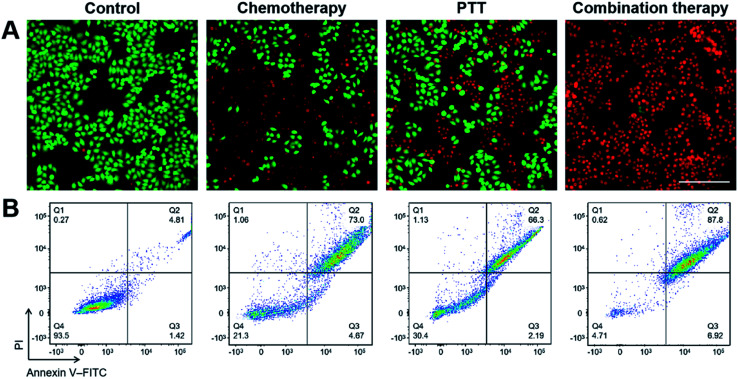
(A) Fluorescence images of HeLa cells after different treatments with calcein-AM/PI staining. The scale bar is 50 μm. (B) Apoptotic analysis of HeLa cells after different treatments with annexin V-FITC/PI staining and detected by flow cytometry.

The *in vitro* antitumor effect of LV–TAX/Au@Ag was first evaluated by fluorescence micrography (Fig. 4A). As HeLa cells were double stained with calcein-AM (green) and PI (red), the green fluorescence represented live cells, while the red fluorescence represented dead cells. Control group showed very high cell viability because HeLa cells in this group were only incubated with PBS without any treatments. There was a certain amount of red fluorescent signals both in chemotherapy group and PTT group, indicating a part of HeLa cells were killed due to chemotherapy or PTT. However, the antitumor effect of a single treatment was not enough. In contrast, almost all of the HeLa cells showed red fluorescence in the group of combination therapy, suggesting an excellent antitumor effect of combination therapy. Moreover, flow cytometry was then used to quantify the antitumor effect of different treatments (Fig. 4B). It showed similar results to that of fluorescence micrography. 93.5% of HeLa cells were alive in control group. On the other hand, 30.4% of HeLa cells were alive in PTT group, while only 21.3% of HeLa cells remained alive after the treatment of chemotherapy due to the direct interaction with TAX. However, in the actual treatment process, it is difficult for all of TAX to contact with tumor cells directly, as some of them may travel to other tissues and be metabolized soon after. Therefore, the antitumor effect of chemotherapy may be greatly reduced *in vivo* treatment. In terms of combination therapy, the cell viability declined to 4.71% with irradiation, which meant that 95.29% of HeLa cells were erased by LV–TAX/Au@Ag, indicating the superior antitumor effect of combination therapy induced by LV–TAX/Au@Ag.

### Bio-distribution and histological study

**Fig. 5:**
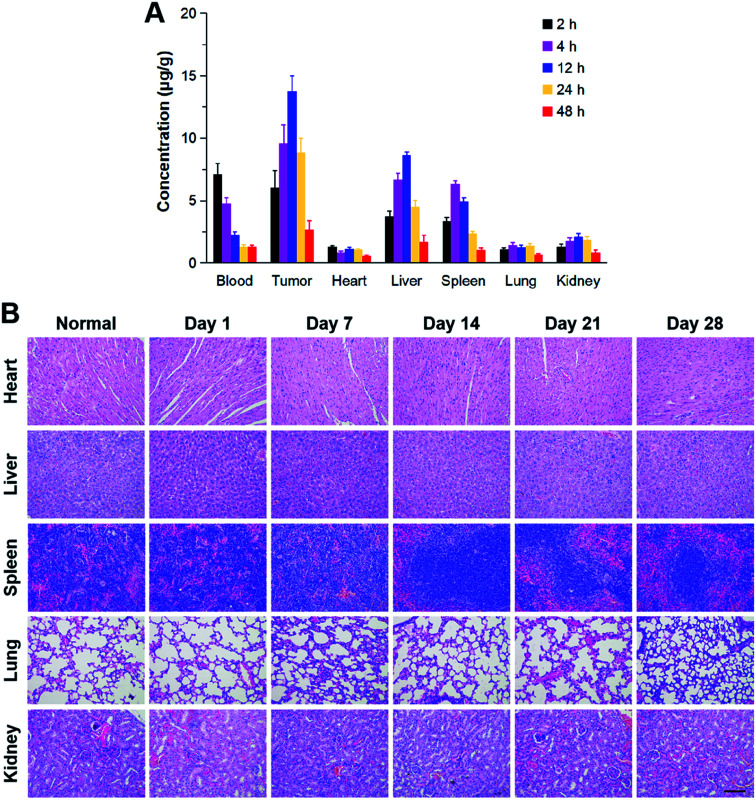
(A) The bio-distribution of LV–TAX/Au@Ag in blood, tumor, and main organs (heart, liver, spleen, lung, and kidney) after intravenous administration. All data represent mean ± SD (*n* = 5). (B) Representative H&E stained sections of main organs after the treatment of LV–TAX/Au@Ag. The scale bar is 50 μm.

In order to verify the targeting ability of LV–TAX/Au@Ag, the bio-distribution of LV–TAX/Au@Ag in HeLa-tumor-bearing mice after intravenous administration was measured by ICP-OES (Fig. 5A). There was a rapid decrease in blood after injection, whereas the concentration of LV–TAX/Au@Ag in liver and spleen rose continuously in the first 12 h post injection, indicating that a certain part of LV–TAX/Au@Ag was taken up by the liver and spleen from the blood circulation. However, the majority of LV–TAX/Au@Ag reached the tumor sites as the concentration in tumor was always higher than that in any other tissues. This result signified LV–TAX/Au@Ag indeed had the capacity to target the tumor through EPR effect.

H&E stained sections of each organ were also taken to evaluate the histological security of LV–TAX/Au@Ag (Fig. 5B). The sections of heart, liver, spleen, lung, and kidney of the mice treated with LV–TAX/Au@Ag were respectively taken at 1, 7, 14, 21, and 28 d post the treatment and dealt with H&E staining. Compared with the images obtained from normal mice, no apparent histological damage was observed in these organs of the treated mice, indicating that LV–TAX/Au@Ag had high biocompatibility and might not cause damage to normal tissues.

### *In vivo* infrared thermal imaging

**Fig. 6:**
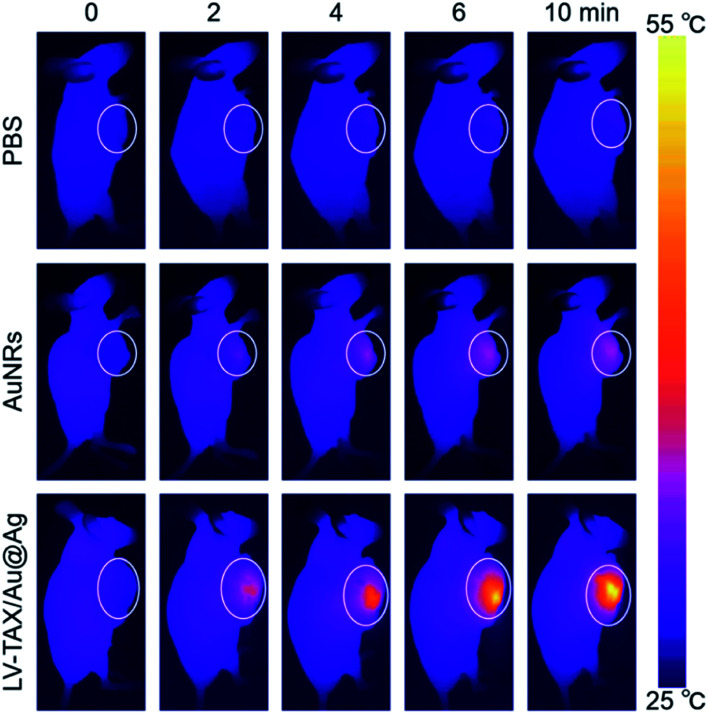
*In vivo* infrared thermal images of HeLa-tumor-bearing mice intravenously injected with PBS, AuNRs, and LV–TAX/Au@Ag respectively after irradiation (808 nm, 1 W cm^−2^).

*In vivo* infrared thermal imaging was carried out to evaluate the photothermal conversion properties of LV–TAX/Au@Ag. The images were taken from the HeLa-tumor-bearing mice that intravenously injected with different materials and irradiated by an 808 nm laser for different exposure time, respectively (Fig. 6). The white circles in the photographs represented the areas of tumors. The temperature of tumor sites in the group of PBS was nearly constant because the simple PBS had no photothermal effect. For the group of AuNRs, the temperature did not rise appreciably due to the lack of tumor targeting ability. As the bare AuNRs had poor water solubility, most of them were directly metabolized instead of reaching the tumor area. However, the distinct increase in temperature was exhibited as the irradiation time increased in the group of LV–TAX/Au@Ag, revealing that LV–TAX/Au@Ag had both capacities of photothermal conversion and tumor targeting. Moreover, Perrault *et al.* have demonstrated that smaller nanoparticles are able to rapidly diffuse throughout the tumor matrix. They observed distinct trends in tumor permeation over the entire size range 8 h post injection, as the 20 nm particles were able to permeate deep into tumor tissues, the 60 nm particles less so, while the most of 100 nm particles were still localized in the perivascular region.^43^ Liu *et al.* also took advantage of this characteristic to construct the pH-responsive polymer-coated gold nanorod clusters. The nanoparticles could be triggered by acidic, leading to a deep tumor penetration effect for enhanced theranostic performance.^44^ Herein, for the group of LV–TAX/Au@Ag, we could also find that the thermal signal was distributed almost throughout the solid tumor, suggesting that the released bare AuNRs were able to penetrate deep into the solid tumor due to their smaller size. Therefore, the tumor cells might be eliminated thoroughly due to the sufficient photothermal effect raised by the bare AuNRs released from LV–TAX/Au@Ag.

### *In vivo* antitumor effect

**Fig. 7:**
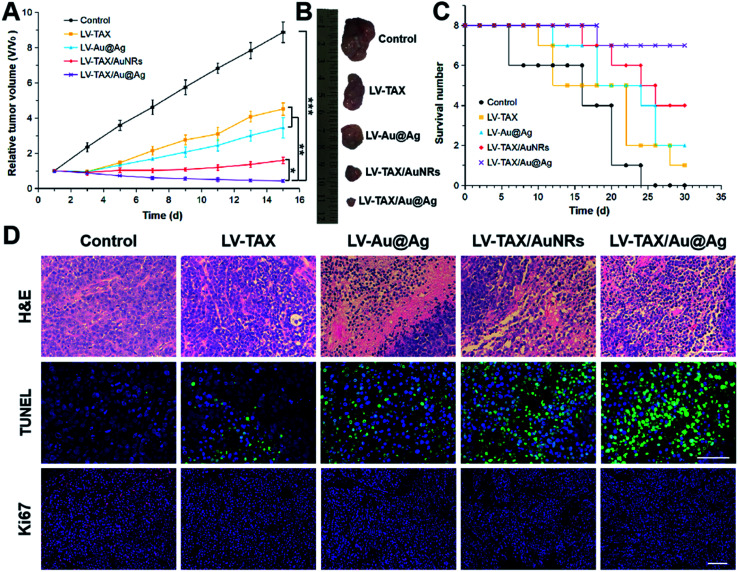
(A) Relative tumor volumes of the HeLa-tumor-bearing mice subjected to various treatments. (B) Representative photographs of tumors collected from the HeLa-tumor-bearing mice subjected to various treatments. (C) Survival number of the HeLa-tumor-bearing mice subjected to various treatments. All data represent mean ± SD (*n* = 8). *p < 0.05, **p < 0.01, ***p < 0.001. (D) H&E, TUNEL, and Ki67 staining on tumor sections of the HeLa-tumor-bearing mice after different treatments. The scale bar is 50 μm.

Considering the advantages of LV–TAX/Au@Ag proved above, the *in vivo* antitumor effect of LV–TAX/Au@Ag was then assessed to demonstrate the therapeutic effect in cancer therapy. Tumor volume and survival number of the mice in different groups were recorded every two days. As displayed in Fig. 7A, tumor grew rapidly in control group and the tumor volume finally reached about 8.86-fold that of the initial day. For groups of LV–TAX and LV–Au@Ag, the tumor growth rate of both was inhibited to some degree in the first few days due to the chemotherapy and PTT, respectively. However, the separate chemotherapy or PTT was not enough to wipe out tumor cells thoroughly. Therefore, the final tumor volume severally increased by 4.51-fold and 3.46-fold compared with the first day. LV–TAX/AuNRs and LV–TAX/Au@Ag exhibited better antitumor effect owing to the combination of chemotherapy or PTT. Nevertheless, the release of TAX and AuNRs in LV–TAX/AuNRs was inhibited while LV–TAX/Au@Ag were able to exhibit a rapid release once reaching the tumor tissues due to the reaction of silver shells and H_2_O_2_. The small sized TAX and AuNRs were then able to penetrate deep into the solid tumor, and fully interact with tumor cells. Finally, tumor volume of LV–TAX/Au@Ag group decreased to 0.42-fold compared with the first day, while that of LV–TAX/AuNRs group reached 1.59-fold. Furthermore, different from LV–TAX/Au@Ag, PTT induced by LV–TAX/AuNRs was non-specific as LV–TAX/AuNRs in normal tissues could also kill cells under near-infrared exposure, which might result in the damage on normal tissues. Therefore, we concluded that LV–TAX/Au@Ag showed the best therapeutic efficacy among all materials. Similarly, the survival number also reflected the outstanding antitumor effect of LV–TAX/Au@Ag, as the most of mice survived at the end of the experiment after treating with LV–TAX/Au@Ag compared with other groups (Fig. 7C).

H&E, TUNEL, and Ki67 staining of tumor sections could further validate the therapeutic effect on tumors (Fig. 7D). Tumor tissues in LV–TAX/Au@Ag group showed a large area of cell deformation, indicating the necrosis and apoptosis of tumor cells, while fewer phenomena occurred in other groups. TUNEL staining showed this kind of contrast more apparently. A large degree of apoptosis (cyan area) could be found in the group of LV–TAX/Au@Ag while the majority of tumor cells stay alive in other groups. In addition, Ki67 staining was used to demonstrate the proliferation. Similarly, LV–TAX/Au@Ag group showed the least positive signals (red fluorescence) compared with other groups, suggesting that the proliferation of tumors was suppressed significantly after the HeLa-tumor-bearing mice were subjected to the combination therapy induced by LV–TAX/Au@Ag.

## Conclusion

In summary, we successfully designed the novel physiological safe and H_2_O_2_-triggered LV–TAX/Au@Ag for combined chemo-photothermal therapy. LV–TAX/Au@Ag were able to target the tumor through EPR effect and take advantage of the silver shells to react with the endogenous H_2_O_2_ in tumor microenvironment. The generated oxygen could disassemble the large LV coated nanosystem into the small sized TAX and AuNRs, which were then able to permeate deep into the solid tumor. The etched bare AuNRs showed the excellent photothermal effect and were able to eliminate the tumor cells with the help of near-infrared laser, while TAX could further suppress the proliferation of tumor cells. The related *in vitro* and *in vivo* experiments proved that the induced combination therapy exhibited an outstanding antitumor efficiency and negligible histological damage. Therefore, we believe that this kind of H_2_O_2_-responsive nanomaterial has broad prospects in future cancer treatment.

## Conflicts of interest

The author reports no conflicts of interest in this work.

## Supplementary Material

RA-010-D0RA04171H-s001
