# Supplementary material for: A tumor-microenvironment-responsive nanomaterial for cancer chemo-photothermal therapy
Source: RSC Adv. 2020 Jun 9;10(37):22091–101. doi: 10.1039/d0ra04171h (PMC9054608; doi:10.1039/d0ra04171h)
Supplement: RA-010-D0RA04171H-s001 [file RA-010-D0RA04171H-s001.pdf]

Electronic Supplementary Material (ESI) for RSC Advances.
This journal is © The Royal Society of Chemistry 2020

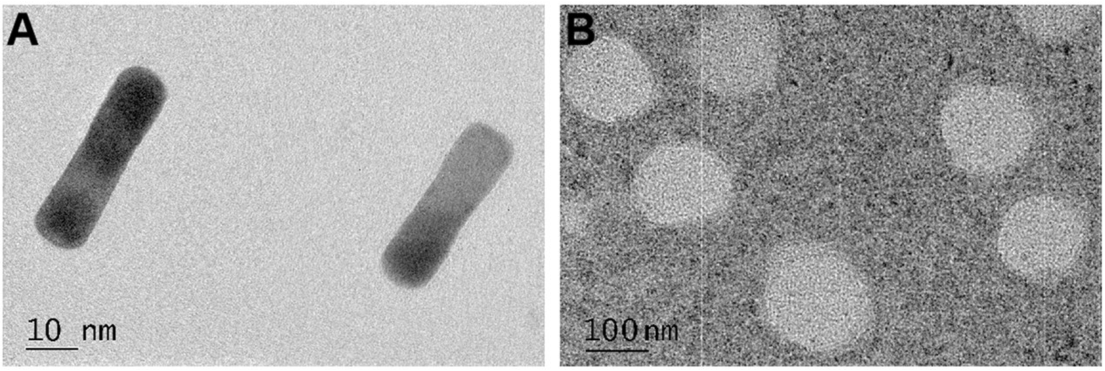

Fig. S1 TEM images of (A) AuNRs and (B) LV that were negative stained with 1 wt% sodium phosphotungstate.

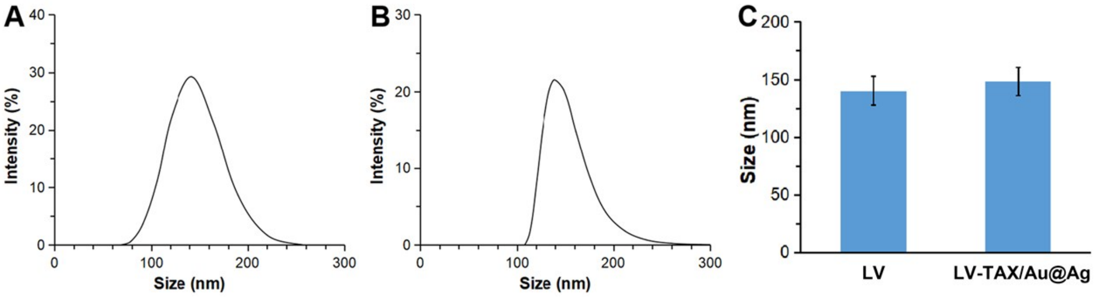

Fig. S2 Size distribution of (A) LV and (B) LV-TAX/Au@Ag. (C) Average diameter of LV and LV-TAX/Au@Ag. All data represent mean ± SD (n = 5).

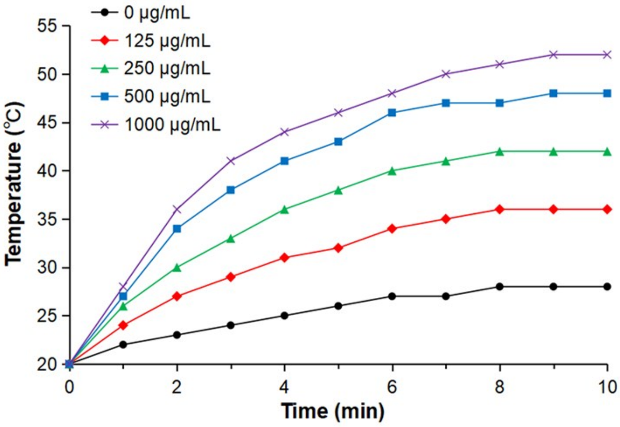

Fig. S3 Increase of temperature at different concentrations of LV-TAX/Au@Ag (etched with H_2O_2) irradiated by an 808 nm laser (1 W/cm^2).
